# Oral health literacy and oral frailty: a parallel mediation model of self-efficacy and health-related quality of life among older adult stroke patients

**DOI:** 10.3389/fpubh.2025.1720294

**Published:** 2025-12-12

**Authors:** Weiwei Yang, Zhenya Liu, Shijuan Gao, Huanyu Li, Guifang Zhang

**Affiliations:** Cerebrovascular Department of Henan Provincial People's Hospital, Henan Provincial Key Medicine Laboratory of Nursing, Zhengzhou University People's Hospital, Zhengzhou, Henan, China

**Keywords:** older adult stroke patients, oral health literacy, oral frailty, oral health self-efficacy, oral health-related quality of life, mediating effect

## Abstract

**Objectives:**

This study aims to investigate the relationship between oral health literacy and oral frailty, as well as the mediating effect of oral health self-efficacy and oral health-related quality of life among older adult stroke patients patients.

**Design:**

A cross-sectional survey.

**Methods:**

Convenience sampling was used to recruit participants, and 298 older adult stroke patients patients completed the General Information Questionnaire, Oral Health Literacy Scale, Oral Frailty Screening Scale, Oral Health Self-Efficacy Scale and Oral Health-Related Impact Scale between October 2024 – March 2025. Structural equation modeling was used to test the relationship between oral health literacy, oral frailty, self-efficacy for oral care, and oral health-related quality of life in older adult stroke patients patients. SPSS 26.0 was used for descriptive statistics and correlation analyses. Structural equation modeling (SEM) was then employed to test the mediating effects.

**Results:**

The mean oral health literacy score among older adult stroke patients patients was 67.74 ± 10.51. oral health self-efficacy averaged 47.84 ± 10.63, while the oral health-related quality of life score was 25.45 ± 8.53. The mean oral frailty score was 4.82 ± 2.77. Oral health literacy was negatively correlated with oral frailty. Both oral health self-efficacy and oral health-related quality of life partially mediated the link between oral health literacy and oral frailty in older adult stroke patients patients.

**Conclusion:**

Oral health self-efficacy and oral health-related quality of life exert parallel mediating effects between oral health literacy and oral frailty. Improving oral health literacy, enhancing self-efficacy, and improving health-related quality of life among older adult stroke patients patients will be important strategies for reducing the risk of oral frailty.

## 1 Introduction

Stroke is not only the second leading cause of death for the older adult(s) worldwide but also the third leading cause of disability and death (1). Its high morbidity, high disability rate, and high mortality rate bring heavy economic and care burdens to society and families (2). Approximately 80% of stroke patients have varying degrees of cognitive, motor, sensory, and other functional impairments, which further limit their ability to maintain oral hygiene (3). Research indicates that approximately 78% of stroke patients experience oral functional disorders (4), primarily manifesting as difficulties in chewing, swallowing, and reduced saliva secretion. These challenges lead to food debris retention, proliferation of pathogenic microorganisms, and an increased risk of oral infections (5). Research shows that stroke patients have worse oral hygiene, more missing teeth, and more severe periodontal disease than non-stroke patients, and also exhibit poorer oral hygiene habits and health beliefs (6). Recognizing this issue, clinical management guidelines for stroke in both Canada and the UK underscore the importance of regular oral health evaluations and strongly recommend integrating effective oral care interventions following a stroke (7, 8). A study from South Korea indicated that poor oral health is positively associated with its risk (9). Poor oral hygiene can lead to various oral diseases, such as bad breath, swollen and bleeding gums, cavities, and periodontitis (10). More critically, it may also cause serious systemic complications including malnutrition and aspiration pneumonia, and even increase the risk of stroke exacerbation, significantly affecting the patient’s quality of life (11). Therefore, maintaining oral health is crucial for the comprehensive management of stroke patients.

Oral frailty is defined as age-related decreases in tooth count, oral hygiene, and oral function, along with decreased interest in oral health, decreased physical and mental reserve capacity, and impaired eating function. This is a state of maladjustment in both physical and psychological aspects (12). Previous studies have shown that in older adults with chronic illnesses, oral frailty increases the risk of negative outcomes, such as malnutrition, systemic frailty, disability, and mortality (13). Additionally, it has a detrimental effect on mental health, causes social disengagement, and lowers quality of life. According to research, the incidence of oral frailty in the older adult(s) rises with age, ranging from 8.1 to 53.2% (14). The prevalence of oral frailty is as high as 47.8% among older stroke patients (15). As a common geriatric syndrome, oral frailty is closely linked to a number of negative outcomes in older patients with ischemic stroke (16). Previous studies have indicated that oral health literacy, oral health self-efficacy, and oral-related quality of life can affect the oral frailty of older adults (17, 18).

Good oral health knowledge, a positive attitude, and proper practices—all components of high Oral Health Literacy (OHL)—can effectively reduce the risk of oral frailty (19). There have already been study proving that oral health literacy is an important predictor of oral frailty (18). Oral Health Literacy (OHL) refers to the ability of individuals to obtain oral health information and knowledge and make correct decisions to maintain their own oral health. Oral health literacy mainly includes knowledge, behavior, skills, and self-efficacy related to oral health (6). It is an important determinant of oral disease prevention and oral health promotion (20). Oral health literacy among the older adult(s) is positively correlated with both the total score and each dimension of oral health self-efficacy (21). The study also found that improving oral health literacy can promote the continuity and stability of oral health behaviors among stroke patients, thereby improving oral health-related quality of life (22). Oral healthcare self-efficacy refers to an individual’s belief in their ability to successfully complete tasks related to oral health. As a pivotal psychological determinant in behavioral change, oral health self-efficacy primarily encompasses knowledge, skills, and behavioral beliefs. However, studies indicate that older adult stroke patients patients in China demonstrate relatively low levels of oral healthcare self-efficacy (23). Critically, a negative correlation exists between oral health-related self-efficacy and oral frailty (24) and is an important influencing factor for the oral frailty (25). Oral health-related quality of life (OHRQoL) is a comprehensive assessment of how oral health affects an individual’s physical, psychological, and social functioning. It directly reflects an individual’s satisfaction with their current oral health status (26). Research showed that older adult stroke patients patients living at home have poor oral health-related quality of life (27). Fortunately, oral health literacy can improve the oral health-related quality of life of older adult stroke patients patients (28).

The Integrated Theory of Health Behaviour Change (ITHBC) was proposed by American clinical nursing expert (29). It is a patient-centred, dynamically focused theoretical model. This model integrates health behavior change theory, self-regulation theory, and social facilitation theory, covering three main patterns and two outcomes. Self-regulation theory is central to the three patterns, while the knowledge-belief pattern and social facilitation pattern form the foundation. This theory suggests that the development and maintenance of healthy behaviors and the improvement of health status are the result of the combined effects of establishing knowledge and beliefs in specific contexts, improving self-management skills, and creating supportive environments. According to ITHBC, an individual’s health literacy (including oral health literacy) directly influences their health behavior and healthy outcomes. When patients have sufficient knowledge and understanding, they are more likely to adopt good oral hygiene practices, which will improve their oral health-related quality of life (OHRQoL). Higher OHRQoL, in turn, affects an individual’s overall health status, including reducing oral frailty. Therefore, this pathway is consistent with the interaction between knowledge, attitudes, and behaviors mentioned in ITHBC. ITHBC emphasizes the importance of self-efficacy in healthy behavior. Higher oral health literacy can enhance patients’ sense of self-efficacy, enabling them to believe in their ability to effectively maintain oral hygiene and actively participate in related activities. Enhanced self-efficacy leads to more effective oral health behaviors, thereby reducing the incidence of oral frailty.

**Figure 1:**
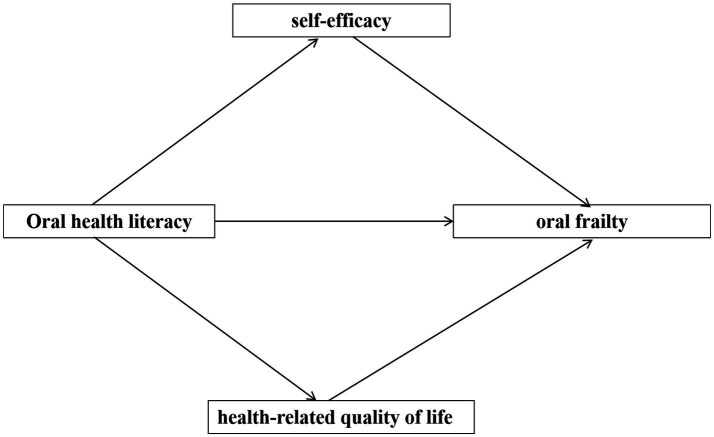
The research framework.

Therefore, based on the Integrated Theory of Health Behaviour Change (ITHBC), oral health self-efficacy and oral health-related quality of life may mediate the relationship between oral health literacy and oral frailty in older adult stroke patients patients. However, existing studies have not systematically explored the underlying mechanisms of these mediating pathways in the older adult stroke patients population. Based on the above theoretical basis and literature review, the research framework as shown in Figure 1. And then we aim to validate the parallel mediating effects of oral health self-efficacy and oral health-related quality of life in the relationship between oral health literacy and oral frailty using structural equation modeling.

## 2 Methods

### 2.1 Research design and participants

This quantitative, cross-sectional study employed an anonymous offline survey from October 2024 – March 2025, utilizing a convenience sample of 298 older adult stroke patients patients from three tertiary hospitals. Inclusion criteria: (1) Patients meeting the stroke diagnostic criteria approved at the 4th National Academic Conference on Cerebrovascular Diseases and confirmed by cranial CT or MRI as having a stroke (30). (2) Age ≥ 60 years; (3) Clear consciousness, no significant cognitive impairment, and able to communicate verbally; (4) All participants provided written informed consent prior to enrollment and participated voluntarily in the study. Exclusion criteria: (1) Patients unable to cooperate with the questionnaire survey; (2) Patients with mental disorders or a family history of mental illness, as such a history may affect the patient’s psychological state and cooperation during the study process.

### 2.2 Sample size calculation

The sample size was calculated using the method proposed by Schoemann et al. (31) for detecting complex mediation effects, via the online tool available at https://schoemanna.shinyapps.io/mc_power_med/. Based on a preliminary survey of 52 older adult stroke patients patients who met the inclusion criteria, the correlation coefficients among oral health literacy, oral health self-efficacy, oral health-related quality of life, and oral frailty were estimated as follows: r₁ = 0.43, r₂ = −0.46, r₃ = −0.66, r₄ = −0.17, r₅ = −0.59, r₆ = 0.41. With a significance level (*α*) of 0.05 and statistical power (1−*β*) of 0.80, the minimum required sample size was 229. To account for an anticipated 30% rate of invalid questionnaires, the final sample size was increased to 298 participants.

### 2.3 Instrumentation

#### 2.3.1 Demographic characteristics

Demographic and clinical characteristics were assessed in this study. The collected data included: age, gender, marital status, education level, smoking status, alcohol consumption, denture use, number of chronic diseases, healthcare payment method, monthly income, and stroke severity (measured by the NIH Stroke Scale, NIHSS).

#### 2.3.2 Oral Frailty Index-8 (OFI-8)

The Oral Frailty Index-8 (OFI-8) is an oral health assessment tool developed by Tanaka et al. (32) through expert consultation and panel discussions. It is designed to identify individuals at risk of oral frailty and functional impairment. The OFI-8 consists of 8 items, with a total score range of 0–11. The OFI-8 comprises eight items, with a total score ranging from 0 to 11. The first three items are scored 2 points for “yes” and 0 points for “no,” while the remaining five items are scored 1 point for “yes” and 0 points for “no.” A total score of 0–3 indicates no oral frailty, and a score of 4 or higher suggests the presence of oral frailty. The scale demonstrates acceptable internal consistency, with a Cronbach’s *α* coefficient of 0.692 in our study. The adapted scale demonstrated good reliability and validity in the Chinese context (33).

#### 2.3.3 Oral Health Literacy Scale (OHLS)

The Oral Health Literacy Scale (OHLS) was developed by Xiang et al. (34). This scale consists of 22 items covering four assessment dimensions: basic oral health knowledge and concepts (6 items), oral-related skills (5 items), oral health behaviors and lifestyle (9 items), and oral health economic support (2 items). It uses a 5-point Likert scale, with scores ranging from 1 (completely disagree) to 5 (completely agree), resulting in a total score of 22–110 points. The score is positively correlated with oral health literacy levels. The specific grading criteria are as follows: extremely low level (22–61points), low level (62–73points), moderate level (74–83 points), and high level (84–110points). The internal consistency reliability of this tool in this study was good (Cronbach’s *α* = 0.807).

#### 2.3.4 Oral Health Self-Efficacy Scale (SESS)

The Oral Health Self-Efficacy Scale (SESS) was originally developed by Kakudate et al. (35). It was later translated into Chinese by Wu et al. (36). It primarily includes three dimensions: oral healthcare visits, proper brushing techniques, and balanced diet, comprising a total of 15 items. It employs a 5-point Likert scale, ranging from ‘completely lacking confidence’ (1 point) to ‘very confident’ (5 points), with a total score range of 15–75 points. Based on self-efficacy levels, it is categorized into three tiers: low level (15–53 points), moderate level (54–59 points), and high level (60–75 points). In this study, the internal consistency reliability of this scale was found to be good (Cronbach’s *α* = 0.903).

#### 2.3.5 Oral Health Impact Profile—14 (OHIP—14)

The Oral Health Impact Profile—14 (OHIP—14) was originally developed by Slade (37). It was subsequently translated and validated for use in Chinese populations by Xin and Ling (38). This scale consists of four dimensions: reduced independence (5 items), psychological distress (3 items), physical functioning (3 items), and pain and discomfort (3 items), totaling 14 items. It uses a 5-point Likert scale, scoring symptoms based on frequency from ‘never’ (0 points) to ‘often’ (4 points), with a total score range of 0–56 points. Scores are negatively correlated with oral health-related quality of life (0–12 points indicate better outcomes, while 13–56 points indicate poorer outcomes). This study demonstrated that the scale has good reliability (Cronbach’s *α* = 0.900).

### 2.4 Data collection

The questionnaire was administered by three trained research nurses at three tertiary-level hospitals. Following nursing administrator approval, participants were recruited in the wards by the 3 trained research nurses, provided with informed consent, and given the questionnaire. They could choose to complete it immediately on-site or within 2 days. (1) Research nurses collected sociodemographic and other data on the day of hospital admission through one-on-one guided questionnaires. For patients who had difficulty completing the questionnaire, researchers filled it out on their behalf by asking questions. (2) Clinical disease data were collected from hospital medical records. Datasets with missing information exceeding 10% were excluded from the study. A total of 298 questionnaires were distributed, and 288 valid questionnaires were returned, yielding a response rate of 96.64%.

### 2.5 Statistical analysis

Statistical analysis was performed using IBM SPSS 26.0 and AMOS 24.0^®^ software (IBM Corporation, Armonk, NY, USA). First, descriptive statistics were computed to characterize the sample, with continuous data presented as mean (± standard deviation) and categorical data as frequencies (percentages). Prior to testing the main hypotheses, common method bias was assessed using Harman’s single-factor test, which indicated it was not a substantial concern in this study. Next, Pearson correlation analysis was conducted to examine the bivariate relationships among the key study variables: oral health literacy, oral frailty, oral health self-efficacy, and oral health-related quality of life. Subsequently, the theoretical model and proposed mediating effects were tested using structural equation modeling (SEM) in AMOS 24.0. The model fit was evaluated against the following criteria: χ^2^/df < 5, RMSEA < 0.08, and RFI, NFI, CFI, TLI, IFI > 0.90 (39). The significance level was set at *p* < 0.05 for all analyses (39).

## 3 Results

### 3.1 Demographic characteristics

The study included 288 participants with a mean age of 68.19 ± 5.88 years (range: 60 ~ 84). The majority were male (56.60%), married (93.06%), and had a monthly income below 3,000 CNY (63.54%). Education levels varied, with36.45% having primary school or lower education. Over half wore dentures (52.78%), and most had at least one chronic disease (95.14%). The primary healthcare payment method was resident medical insurance (58.34%). One hundred and seventy six older adult stroke patients patients experienced oral frailty, with an incidence rate of 61.1%. Additionally, 60.07% of participants had mild stroke symptoms based on NIHSS scores (Table 1).

**Table 1:** Demographic characteristics (*N* = 288).

Characteristics	Classification	n (%)/M ± SD
Age	60 ~ 84	68.19 ± 5.88
Gender	Male	163 (56.60%)
	Female	125 (43.40%)
Marital status	Married	268 (93.06%)
	Unmarried/divorced/separated	20 (6.94%)
Education level	Primary school or lower	105 (36.45%)
	Junior high school	90 (31.25%)
	Senior high school	70 (24.31%)
	Junior college or above	23 (7.99%)
Smokers	Yes	103 (35.80%)
	No	185 (64.20%)
Drinkers	Yes	93 (32.30%)
	No	195 (67.70%)
Dentures	Yes	152 (52.78%)
	No	136 (47.22%)
Chronic diseases	0	14 (4.86%)
	1	80 (27.78%)
	2	105 (36.46%)
	≥3	89 (30.90%)
Healthcare payment method	Self-funded	5 (1.74%)
	Resident medical insurance	168 (58.33%)
	Employee medical insurance	83 (28.82%)
	Government-funded medical insurance	32 (11.11%)
Monthly income	<3,000	183 (63.54%)
	3,000 ~ 5,000	73 (25.35%)
	>5,000	32 (11.11%)
NIH Stroke Scale (NIHSS)	Mild stroke	173 (60.07%)
	Moderate to severe	115 (39.93%)

### 3.2 Mean and correlation between major variables

The means of oral health literacy, oral frailty, oral health self-efficacy and oral health-related quality of life were 67.74 ± 10.51, 4.82 ± 2.77, 47.84 ± 10.63 and 25.45 ± 8.53, respectively. The correlation effect of the four variables are shown in Table 2. There is a significant positive correlation between oral health literacy and oral health self-efficacy (r = 0.465, *p* < 0.01), while both variables show significant negative correlations with oral health-related quality of life (r = −0.467 and r = −0.325 respectively, *p* < 0 0.01) and oral frailty (r = −0.612 and r = −0.547 respectively, *p* < 0.01). Additionally, oral health-related quality of life demonstrates a significant positive correlation with oral frailty (r = 0.475, *p* < 0.01).

**Table 2:** Descriptive statistics and bivariate correlations of the main study variables (*N* = 288).

Variables	Mean	SD	1	2	3	4
1. Oral health literacy	67.74	10.51	1			
2. Oral health self-efficacy	47.84	10.63	0.465**	1		
3. Oral health-related quality of life	25.45	8.53	−0.467**	−0.325**	1	
4. Oral frailty	4.82	2.77	−0.612**	−0.547**	0.475**	1

M, mean; SD, standard deviation.**P < 0 0.01 (two-tailed).

### 3.3 Parallel mediating effect

**Figure 2:**
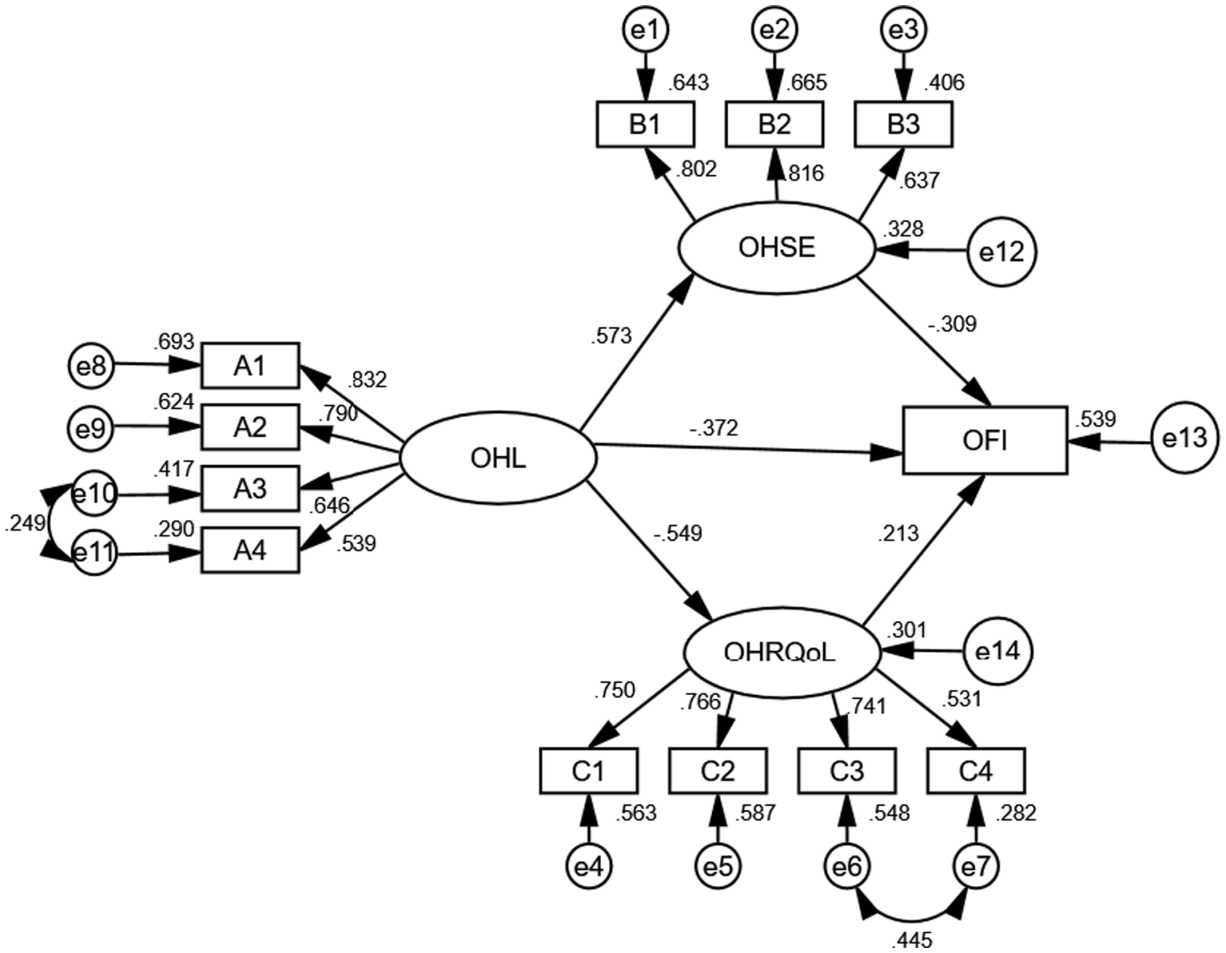
Structural equation model for the parallel-mediating effect. A1: Healthy lifestyle and behaviors. A2: Basic oral health knowledge and concepts. A3: Oral health-related skills. A4: Willingness to provide financial support. B1: Self-efficacy for regular dental visits. B2: Self-efficacy for proper tooth brushing. B3: Self-efficacy for balanced diet. C1: Decline in independent functioning. C2: Psychological discomfort. C3: Physical functional impairment. C4: Oral pain and discomfort.

Common method bias was assessed via Harman’s single-factor test; the first unrotated factor explained only 18.43% of the variance [below the 50% threshold (40)] across all 59 items, indicating it was not a significant concern. A structural equation model was established. As shown in Figure 2, the measurement model was acceptable. χ^2^/df = 2.551, RMSEA = 0.074 < 0.08, IFI = 0.950, CFI = 0.949, TLI = 0.930. Table 3 shows that the total effect of oral health literacy on oral frailty was −0.666, 95% CI [−1.013 ~ −1.211]. The direct effect of Oral health literacy and oral frailty was −0.372, 95% CI [−0.835 ~ −0.304]. The total indirect effect of oral health literacy and oral frailty was −0.294, 95% CI [−0.687 ~ −0.263].

**Table 3:** Mediating effects analysis.

Effect	β	SE	95% CI	*P*	Proportion of total effect (%)
Direct: OHL → OFI	−0.372	0.135	−0.835 ~ −0.304	<0.01	55.85%
Indirect1: OHL → OHSE → OFI	−0.177	0.077	−0.449 ~ −0.137	<0.01	26.58%
Indirect2: OHL → OHRQoL → OFI	−0.117	0.071	−0.337 ~ −0.054	<0.01	17.57%
Total indirect effect of OHL on OFI	−0.294	0.106	−0.687 ~ −0.263	<0.01	44.15%
Total effect of OHLS on OFI	−0.666	0.093	−1.013 ~ −1.217	<0.01	1

CI, confidence interval; SE, Standard Error.

## 4 Discussion

This study results not only validated the direct effects of self-efficacy on oral health-related quality of life but also revealed the parallel mediating roles of oral health literacy and oral frailty in this relationship.

The incidence of oral frailty in this study (61.1%, 176/298) was higher than that reported in Wuhu (15) and Guangdong (41). but lower than the rate found at the Second Affiliated Hospital of Anhui University of Chinese Medicine (42). These discrepancies may stem from differences in population characteristics (e.g., age, stroke severity); or from variations in interventions like oral care, nutrition, and rehabilitation. Notably, the higher incidence rate (66.7%) reported in the study from the Second Affiliated Hospital of Anhui University of Chinese Medicine might be due to the inclusion of more severe cases or a higher proportion of older adult(s) patients (42). These factors collectively account for the variations in oral frailty incidence rates observed across different studies. Consequently, future research should focus on standardizing assessment protocols and strengthening multicenter collaboration to generate more representative and generalizable data.

The structural equation modeling results demonstrate that oral health literacy directly predicts oral frailty in older adult stroke patients patients (*β* = −0.372, 95% CI: −0.835 to −0.304, *p* < 0.01). This effect accounted for 55.85% of the total predictive strength observed in the model. This finding aligns with the work of (43), whose research in an older adult(s) population similarly demonstrated a significant inverse relationship between oral health literacy and oral frailty. The higher the oral health literacy, the lower the risk of oral frailty. Another study by Xiao et al. (18) further demonstrated that ischemic stroke patients with limited health literacy face an elevated risk of oral frailty and require multidimensional interventions to modify their health behaviors. Good oral health knowledge, a positive attitude, and proper practices can effectively reduce the risk of oral frailty (19).

What makes us even more delighted is that the results demonstrated a significant pathway whereby oral health literacy reduces oral frailty risk by enhancing patients’ oral health self-Efficacy. This partial mediation effect of oral health self-efficacy (*β* = −0.177, 95% CI: −0.449 to −0.137, *p* < 0.01) explained 26.58% of the total variance, This result further confirms our research hypothesis. A comprehensive understanding of oral health knowledge enhances individuals’ confidence in performing standard oral care behaviors, such as proper brushing and flossing (44). Moreover, studies have also found that patients with high levels of oral self-efficacy have a lower risk of oral frailty (45). Grounded in social cognitive theory (46), oral health literacy empowers older adult stroke patients patients by fostering the self-efficacy necessary for maintaining oral hygiene. This enhanced confidence translates into sustained health behaviors—such as regular cleaning and dental check-ups—which ultimately mitigates the risk of oral frailty. Older adults with low self-efficacy show increased susceptibility to various oral health issues (47). These problems—including gingival bleeding, periodontitis, dental plaque, and dental caries—significantly elevate their risk of developing oral frailty. Due to hemiplegia-induced impairment of cleaning ability and neuropsychological disorders (e.g., unilateral neglect and anosognosia), stroke patients often exhibit markedly reduced compliance with oral care and diminished ability to recognize oral health problems. These challenges heighten the significance of self-efficacy, which plays an increasingly critical mediating role in the progression of oral frailty. The compounded impact of motor and cognitive deficits further amplifies the influence of self-efficacy in this population. These findings underscore the necessity of self-efficacy-focused, personalized interventions.

What was even more surprising to us was that oral health-related quality of life (OHRQoL) serves as a partial mediator between oral health literacy and oral frailty among older adult stroke patients patients. The mediating effect is significant (*β* = −0.117, 95% CI: −0.337 to −0.054, *p* < 0.01) and explains 17.57% of the total variance. In this model, OHRQoL played a relatively important mediating effect. First, the findings of this study are consistent with those reported by Babadi et al. (48) in patients with bladder cancer. Higher oral health literacy is associated with a smaller negative impact on OHRQoL (49). Higher health literacy can improve oral and dental health as well as OHRQoL in older adult stroke patients patients by enhancing awareness, increasing access to healthcare services, and boosting the frequency of dental visits. Second, OHRQoL can directly influence the progression of oral frailty in older adult stroke patients patients. The study by Puranen et al. (28) further supports this perspective, emphasizing the importance of OHRQoL in oral frailty among the older adult(s). OHRQoL serves as a sensitive and multidimensional indicator that effectively captures the spectrum of patients’ subjective experiences, including functional limitations (e.g., mastication difficulties), communication challenges, and psychosocial impacts such as social embarrassment (26). A decline in OHRQoL itself is a manifestation of reduced oral function and discomfort. Therefore, OHRQoL can serve as an early warning signal. By improving the oral health literacy of older adult stroke patients patients, their OHRQoL can be enhanced, indirectly predicting the development of oral frailty.

Finally, this study highlights a crucial finding: oral health self-efficacy and oral health-related quality of life play a synergistic role as parallel mediators in the relationship between health literacy and oral frailty among older adult stroke patients patients. This finding aligns with the Integrated Theory of Health Behavior Change (ITHBC), which conceptualizes health literacy as a key factor that promotes behavior change through empowerment and perception. Our results embody this framework by revealing two distinct parallel pathways through which oral health literacy operates. In the “Empowerment-Behavior” pathway, high literacy enhances patients’ confidence (self-efficacy) in performing oral care behaviors by improving their skills and knowledge; this in turn motivates them to overcome barriers and adhere to health behaviors, directly maintaining oral function and delaying oral frailty (47). In the “Experience-Motivation” pathway, high literacy promotes behavior change, effectively reducing oral pain and functional impairment, thereby improving quality of life across physical, social, and psychological dimensions; this improvement in quality of life serves as an intrinsic incentive that reinforces long-term motivation, indirectly halting the progression of oral frailty (50). Clinically, these results advocate for a dual-path intervention strategy. It is essential to not only build self-management confidence through skill training but also to improve life quality by alleviating symptoms and restoring function. This complementary approach accounts for the independent and synergistic mechanisms—whereby self-efficacy promotes health behaviors that enhance quality of life, creating a positive feedback loop—to effectively mitigate oral frailty risk.

## 5 Limitations

This study has the following limitations that warrant attention: First, the cross-sectional design limits the findings to correlational inferences, thereby requiring longitudinal studies to establish causality. Second, the reliance on self-report questionnaires leaves the study prone to recall bias, incorporating objective measures like clinical oral examinations is needed to enhance validity. Additionally, all study participants were from three general hospitals in the same region, which may limit the representativeness of the sample.

## 6 Conclusion

The results indicate that oral health literacy not only directly predicts the occurrence and development of oral frailty but also indirectly influences it through parallel mediating pathways involving oral health self-efficacy and oral-related quality of life. This finding deepens the existing understanding of the mechanisms underlying the effects of oral health literacy and provides new theoretical foundation and practical directions for interventions targeting oral frailty in stroke patients.

## Data Availability

The raw data supporting the conclusions of this article will be made available by the authors, without undue reservation.
